# 17β-Oestradiol treatment modulates nitric oxide synthase activity in MDA231 tumour with implications on growth and radiation response

**DOI:** 10.1038/sj.bjc.6600032

**Published:** 2002-01-07

**Authors:** E C Chinje, K J Williams, B A Telfer, P J Wood, A J van der Kogel, I J Stratford

**Affiliations:** Experimental Oncology Group, School of Pharmacy and Pharmaceutical Sciences, University of Manchester, Oxford Road, Manchester M13 9PL, UK; Department of Pharmacy and Pharmacology, University of Bath, Claverton Down, Bath BA2 7AY, UK; Institute of Radiotherapy, University Hospital Nijmegen 6525 GA Nijmegen, The Netherlands

**Keywords:** nitric oxide synthase, 17β-oestradiol, breast tumour, hypoxia, radiation response

## Abstract

The putative oestrogen receptor negative human breast cancer cell line MDA231, when grown as tumours in mice continually receiving 17β-oestradiol, showed substantially increased growth rate when compared to control animals. Further, we observed that 17β-oestradiol treatment could both increase the growth rate of established MDA231 tumours as well as decreasing the time taken for initiating tumour growth. We have also demonstrated that this increase in growth rate is accompanied by a four-fold increase in nitric oxide synthase activity, which was predominantly the inducible form. Inducible-nitric oxide synthase expression in these tumours was confirmed by immunohistochemical analysis and appeared localized primarily in areas between viable and necrotic regions of the tumour (an area that is presumably hypoxic). Prophylactic treatment with the nitric oxide synthase inhibitor nitro-L-arginine methyl ester resulted in significant reduction in this apparent 17β-oestradiol-mediated growth promoting effect. Tumours derived from mice receiving 17β-oestradiol-treatment were characterized by a significantly lower fraction of perfused blood vessels and an indication of an increased hypoxic fraction. Consistent with these observations, 17β-oestradiol-treated tumours were less radio-responsive compared to control tumours when treated with a single radiation dose of 15 Gy. Our data suggests that long-term treatment with oestrogen could significantly alter the tumour oxygenation status during breast tumour progression, thus affecting response to radiotherapy.

*British Journal of Cancer* (2002) **86**, 136–142. DOI: 10.1038/sj/bjc/6600032 www.bjcancer.com

© 2002 The Cancer Research Campaign

## 

Nitric oxide (NO) acts as an intercellular secondary messenger in all mammalian organs, participating in a host of functions including vascular homeostasis. It is synthesized from L-arginine by a family of isoenzymes called NO synthases (NOS) (Nathan, 1992). However, its role in tumour biology is complex and still poorly understood. NO is involved in regulating tumour cell growth by acting as part of a signalling cascade for neovascularization *in vivo* (Jenkins *et al*, 1995). NO has also been demonstrated to affect the transcription of certain genes, including the vascular endothelial growth factor (VEGF) which is the basis for angiogenesis associated with tumour growth and metastasis (Folkman, 1995). Growth of solid tumours is regulated by interactions of several cell types including those of the tumour vasculature, infiltrating immune cells, such as macrophages and the tumour cells themselves (Sutherland *et al*, 1988). It has been shown that elevated levels of NOS are present in human tumours when compared to surrounding normal tissue and further that expression is related to tumour grade (Thomsen *et al*, 1994, 1995; Cobbs *et al*, 1995).

Previous studies within our laboratories involved manipulating levels of NO production and hence tumour oxygenation status in order to gain some understanding of the biochemical and molecular consequences that the presence of hypoxia imposes on the biology of solid tumours. We were able to demonstrate that administration of NOS inhibitors to tumour-bearing mice led to a significant decrease in tumour oxygenation and increase in radiation resistance (Wood *et al*, 1994a,b). Other workers have used inhibitors of NOS activity to decrease tumour blood flow (Andrade *et al*, 1992) and retard the growth of some experimental tumours (Thomsen *et al*, 1997).

Oestrogens are well known to regulate the growth and development of normal human mammary tissue (Topper and Freedman, 1980). They have also been implicated in promoting the growth of most oestrogen-receptor (ER) positive mammary carcinomas through their mitogenic effects on the cells via these receptors (McGuire *et al*, 1975; Dickson and Lippman, 1987). It is proposed that 17β-oestradiol (E_2_) enters the target cells and binds to nuclear ER. Following association of the hormone-ER complex with specific DNA sequences (oestrogen response elements), the transcription of a specific set of genes is initiated, leading, among other events, to increased proliferation. This dependence has been well documented with the breast tumour cell line, MCF-7, both *in vitro* and *in vivo* (Shafie and Grantham, 1981). Conversely, MDA231 represents an example of a breast cancer cell line that has been shown not to transcribe the ER gene (Weigel and deConnick, 1993). Studies carried out by Friedl and Jordan (1994), using a subclone of MDA231 tumour cells, demonstrated that E_2_ had no effect on the growth of these cells *in vitro* but stimulated growth *in vivo*, thereby suggesting an animal host-mediated mechanism of action was most likely. NK cells are lymphocytes that rapidly kill certain tumour cells and are believed to play an important role in controlling metastases (Kozlowski *et al*, 1984; Shakhar and Ben-Eliyahu, 1998). In addition, these studies as well as that reported by Seaman *et al* (1978) have linked suppression of NK activity with chronic administration of E_2_. However, the precise role of E_2_ in suppressing NK cell activity is still very debatable. For instance, by using a strain of immunodeficient mice that does not have NK cells, the findings of Friedl and Jordan (1994) argued strongly against NK cell-mediated mechanism of action by E_2_. A similar view was shared by other workers who could not demonstrate a correlation between growth control of human tumours and NK cell activity in athymic mice with different immune effects (Fodstad *et al*, 1984). There is now accumulating evidence demonstrating that E_2_-treatment can increase the expression of NOS in a wide range of tissues (Cobbs *et al*, 1995; Thomsen *et al*, 1995; Weiner *et al*, 1994). In this study, we have investigated the effect of long term E_2_-treatment on the expression of NOS activity in the putatively ER-negative human xenograft MDA231 and propose that NO is an important mediator in promoting this increase in tumour growth rate. In addition, we have provided some evidence that demonstrates E_2_-treatment may alter the oxygenation status of this tumour type and consequently its radiation response.

## MATERIALS AND METHODS

### Materials

17β-oestradiol pellets (1.7 mg, 60-day release) were purchased from Innovative Research of America (Sarasota, USA). NADPH, Dowex-50WX8-400 and N^ω^-nitro-L-arginine methyl ester (L-NAME) were obtained from Sigma (Poole, UK). Tissue culture medium was obtained from GIBCO-BRL (Paisley, UK) and foetal calf serum was bought from PAA Laboratories (Wiener Strasse, Austria). All other reagents were of analytical grade and were purchased from Sigma (Poole, UK) or otherwise as indicated in the text.

### Cell culture

The MDA231 cells were maintained in exponential growth phase in RPMI-1640 medium supplemented with 2 mM glutamine and 10% (v/v) foetal calf serum, in an atmosphere of 5% CO_2_ in humidified air at 37°C.

### Growth of tumour xenografts

All animal procedures were carried out in accordance with the Scientific Procedures Act 1986 and in line with the UKCCCR guidelines 1999, by approved protocols (Home Office Project Licence No. 40-1770).

#### Effect of 17*β*-oestradiol treatment

Ten female nu/nu mice were injected subcutaneously with 2×10^6^ MDA231 cells on the back in a volume of 0.1 ml and allowed to grow to about 200 mm^3^ in volume. Half of animals with size-matched tumours were then treated with E_2_ pellets (1.7 mg, 60 days release) implanted in the scruff of the neck and growth measurements were taken until tumours attained a volume of about 600 mm^3^. In another set of five animals, E_2_-treatment was administered 2 days prior to tumour implantation in order to investigate its effect on the onset of tumour growth. Measurements were again taken until tumours attained 600 mm^3^. All tumours were excised once they attained the required growth size and either fixed or snap frozen in liquid nitrogen for subsequent analysis.

#### Effect of L-NAME treatment

L-NAME was stored frozen at −20°C and added to drinking water and administered *ad libitum* (1 mg ml^−1^) to 10 mice. Treatment commenced 2 days prior to tumour implantation and continued while growth was monitored until tumours reached 600 mm^3^. Half of these animals also received E_2_ pellets (1.7 mg, 60-day release) implanted on the scruff of the neck.

### NOS activity measurements

Snap frozen tumours were thawed and homogenized (Ultra-Turrax T25 homogenizer) in four volumes of ice-cold buffer containing HEPES (10 mM, pH 7.4), sucrose (320 mM), EDTA (100 μM), dithiothreitol (0.05 mM), leupeptin (10 μg ml^−1^), soybean trypsin inhibitor (10 μg ml^−1^) and aprotinin (2 μg ml^−1^). The preparations were then sonicated using an MSE Soniprep 150 for 3×5 s at a nominal frequency of 23 kHz and an oscillation amplitude of between 5 and 10 μm. Samples were placed in ice between each sonication. These suspensions were allowed to stand in ice for a further 10 min, and then centrifuged at 9000 **g** for 15 min at 4°C. The resultant pellet was discarded and the post-mitochondrial supernatant (cytosol and microsomes) was treated with a strong cation exchange resin (Dowex-50WX8-400) to remove endogenous arginine. The supernatant was incubated with the resin for 5 min and centrifuged at 9000 **g** for 5 min in order to pellet the resin. This process was repeated twice, after which the cytosol was treated as free of endogenous arginine.

Nitric oxide synthase activity was measured by monitoring the conversion of L-[*U*-^14^C]-arginine to L-[*U*-^14^C]-citrulline. The reaction mixture (final volume 150 μl) consisted of HEPES buffer (20 mM, pH 7.4), L-valine (50 mM), L-citrulline (100 μM), 10 μM L-arginine and 50 μCi ml^−1^ l-[*U*-^14^C]-arginine, tetrahydrobiopterin (10 μM), calmodulin (400 U ml^−1^), dithiothreitol (2.5 mM), calcium chloride (250 μM), bovine serum albumin (75 mg ml^−1^) and 1 mM NADPH. The reaction was initiated by the addition of 50 μl of tumour extract (100–300 μg protein) and incubated at 37°C for 10 min. The reaction was terminated by the addition of 5 ml of 50% (v/v) Dowex-50WX8-400 resin in water to bind any remaining arginine. The resin-incubate mix was then left to settle for 20 min before taking an aliquot of the supernatant for analysis by scintillation counting. The enzyme activity associated with iNOS (calcium-independent activity) was measured as a difference in activity carried in the absence and presence of 1 mM ethylene-bis-(oxyethylenenitrilo) tetra-acetic acid (EGTA).

### Oestrogen receptor (ER) determination

ER determination was carried out in tissue cytosol by employing a monoclonal antibody kit (ABBOTT ER-EIA Monoclonal, Abbott Laboratories, Diagnostic Division, Abbott Park, IL, USA). The sensitivity of the system is calculated as the concentration of ER that was distinguishable from the zero standard, i.e., two standard deviations above the zero standard. This was found to correlate to 1.5 fmol ER mg protein^−1^ for a cytosol that is 1 mg protein ml^−1^.

### Immunohistochemistry

Tumours were excised and immediately fixed to provide optimum antigen retrieval and paraffin-embedded for immunohistochemical analysis. Sections were stained with haemotoxylin for cell nuclei. To visualize iNOS localization on sections, an anti-iNOS polyclonal antibody (TCS Biologicals Ltd, Bucks, UK) was employed at a dilution of 5 μg ml^−1^. After washing off excess primary antibody, this was followed by incubation with a biotinylated bridging antibody and an avidin-biotin complex labelled horseradish peroxidase (Dako Ltd., High Wycombe, Bucks, UK). Following incubation with 3,3-diaminobenzidine hydrochloride (DAB, Sigma, Poole, UK) as a substrate, positive staining for the presence of iNOS was seen as a dark brown end product.

### Analysis of tumour hypoxia, vessel density and perfusion

Tumours that had reached 200 mm^3^ in volume from control and E_2_-treated groups were treated with 7-(4′-(2-nitroimidazole-1-yl)-butyl)-theophylline (NITP) 2 h prior to sacrifice (140 mg kg^−1^ in peanut oil containing 10% DMSO). Hoechst 33342 was next administered 1 min prior to sacrifice (20 mg kg^−1^ in PBS, i.v. via the tail vein). Mice were culled, tumours rapidly excised, snap frozen and stored at −80°C.

Immunohistochemical staining and subsequent analytical procedures were carried out as described elsewhere (Bussink *et al*, 1998). Briefly, 5-μm sections were scanned for Hoechst 33342 signal then treated overnight at 4°C with rabbit-anti NITP (anti-theophylline, Sigma, Poole, UK) diluted 1 : 10 in 9F1 supernatant (rat anti-mouse endothelium antibody). Sections were then treated simultaneously with biotinylated-donkey anti-rabbit antibody and tetramethylrhodamine isothiocyanate (TRITC)-conjugated goat anti rat antibody (Jackson Immunoresearch Laboratories, PA, USA) for 1 h at room temperature. Finally, ALEXA488-conjugated streptavidin (Molecular Probes, Leiden, The Netherlands) and TRITC-conjugated donkey anti-goat antibodies were applied for another 1 h at room temperature. Hypoxic regions and vessel signals were then scanned to overlay the initial Hoechst image and composite binary images produced. Vessel density was calculated from the total number of vessels divided by the tumour area (excluding any appreciable areas of necrosis, determined from haematoxylin and eosin staining). Perfused vessels were identified as those where Hoeschst 33342 and 9F1 signals overlapped. The perfused fraction (PF) was calculated by dividing the area of perfused vessels by the total vascular area.

### Radiation treatment

Mice were restrained but unanaesthetized during this procedure. Control and E_2_-treated tumours that had attained 200 mm^3^ were locally irradiated with a single dose of 15 Gy delivered at 2 Gy min^−1^ and allowed to grow to three times the size at the time of treatment. The time taken for each tumour to double in volume from treatment size (TD) was obtained from the log phase of growth. In all treatment groups, regression analysis to calculate r^2^ values confirmed a linear relationship. To make statistical comparisons between the response of the tumours with or without E_2_-treatment, the specific growth delay (SGD) was calculated for each tumour using the following equation:


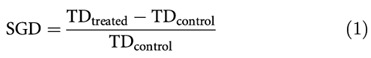


TD_control_ represents the initial doubling time of the untreated tumours calculated from growth rate attained during exponential growth phase for each tumour type (Bailey *et al*, 1980; Kopper and Steel, 1975). Hence, the SGD values represent the number of TDs saved by the radiation treatment.

### Statistical analysis

All the numerical data from analysis between the different treatment groups were expressed as mean value±1 standard deviation (s.d.). Tumour doubling times (TD) were calculated from the log phase and linear regression analysis was performed to confirm a linear relationship. Statistical comparisons of differences in mean tumour measurements after treatment between groups were carried out using a two-way analysis of variance (ANOVA) followed by an unpaired Student's *t*-test. Significance was achieved if *P*<0.05.

## RESULTS

### Effect of E_2_-treatment on tumour growth rate and NOS activity

**Figure 1:**
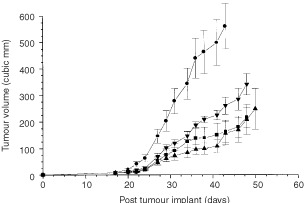
Growth of MDA231 breast tumours in mice. Animals were treated with E_2_ (•) (1.7 mg, 60-day sustained release pellets), E_2_ plus L-NAME (▾), L-NAME alone (▴) and no treatment (▪). Tumour doubling (TD) times from these treatment groups were obtained from the log phase of growth. Calculated r^2^ values from linear regression analysis were 0.981, 0.984, 0.953 and 0.995 for control, E_2_-treatment alone, E_2_-treatment with L-NAME, and L-NAME alone, respectively.

**Figure 2:**
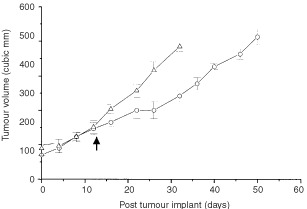
Effect of E_2_ on growth of established MDA231 breast tumours. Tumours were allowed to grow to approximately 150 mm^3^ and half of sized-matched tumours were either treated with E_2_ (▵) (1.7 mg, 60-day sustained release pellets) or left untreated (○). ↑ indicates point at which E_2_-treatment was commenced.

The effect of E_2_-treatment on the growth of MDA231 tumours was studied either with treatment commencing prior to tumour implantation (Figure 1

) or on matched tumours that had attained a size of about 200 mm^3^ (Figure 2

). E_2_-treatment prior to tumour implantation significantly enhanced the tumour growth rate with TD values of 7±0.7 days compared to 12±1.3 days for control tumours (Table 1

**Table 1:**
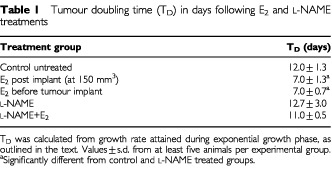
Tumour doubling time (T_D_) in days following E_2_ and L-NAME treatments

). When E_2_-treatment was administered to mice bearing established tumours, the growth rate of these tumours were again increased (TD of 7.0±1.3 days compared to 12±1.3 days for control). Hence, E_2_-treatment did not only increase the onset of tumour growth but also increased the growth rate of established tumours. This increase in tumour growth rate following E_2_-treatment was accompanied by at least a 4–5-fold induction of iNOS activity as determined by the conversion of L-[^14^C]-arginine to L-[^14^C]-citrulline (Table 2

**Table 2:**
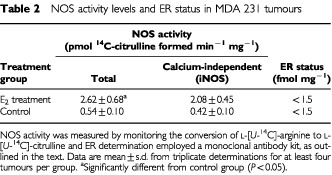
NOS activity levels and ER status in MDA 231 tumours

).

### Effect of L-NAME treatment on tumour growth

Also represented in Figure 1 (and included in Table 1) is growth delay data following prophylactic treatment with L-NAME. A relationship was apparent between NOS activity expression and tumour growth. Treatment with L-NAME abolished the growth promoting effect by E_2_ (Figure 1). This was evident from TD values of 12.7±3.0 and 11.4±0.5 days for L-NAME treatment alone or in combination with E_2_, respectively and these values were not different from those of untreated tumour-bearing mice (12.0±1.3 days).

### ER measurements in xenografts

Results obtained from ER measurements showed levels for both control and E_2_-treatment groups were below the sensitivity limits of the monoclonal system used which was 1.5 fmol ER mg protein^−1^ (Table 2). Based on this information, tumours obtained from both experimental groups were judged to be ER-negative.

### Immunohistochemistry

**Figure 3:**
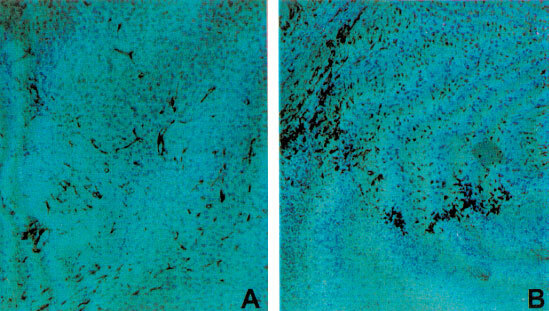
*In situ* localization of iNOS in MDA231 breast tumour. A primary anti-iNOS polyclonal antibody was applied on control (**A**) and E_2_-treated (**B**) tumour sections (for details see Materials and methods'). Haematoxylin was used as a nuclear counterstain. Following incubation with a biotinylated bridging and an avidin-biotin complex labelled horseradish peroxidase, positive staining for the presence of iNOS was seen as a dark brown end-product after incubation with 3,3-diaminobenzidine hydrochloride (DAB) as a substrate.

Confirmation of *in situ* localization of NOS in tumours utilized a rabbit polyclonal antibody for iNOS. The results obtained (Figure 3

) show positive staining for NOS across all treatment groups particularly in the endothelium of blood vessels and some connective tissue cells. A weaker staining of tumour cells was observed in controls. However, in the E_2_-treatment group, there was consistent localisation of NOS expression (intense staining) in a region between ‘viable’ tumour cells and true necrotic regions.

### Analysis of tumour hypoxia, vessel density and perfusion

Multiparameter analysis for tumour hypoxia, vessel density and extent of perfusion were carried out on tumours that had reached 200 mm^3^. The results obtained (Table 3

**Table 3:**
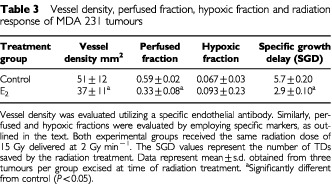
Vessel density, perfused fraction, hypoxic fraction and radiation response of MDA 231 tumours

) suggested that E_2_-treated tumours presented with an elevated hypoxic fraction and lower vessel density when compared to those in the control group. In addition, the E_2_-treated tumours were characterized by a lower fraction of perfused vessels. Despite these trends, the difference in hypoxic fraction between control and E_2_-treated tumours was found not to be statistically significant at 5% level of significance (*P*<0.05).

### Tumour radiation response

To assess the therapeutic consequences of the altered growth response by E_2_-treatment, a single radiation dose of 15 Gy was administered to size-matched tumours at 200 mm^3^ in size. SGD data was calculated for each treatment group when tumours attained three times treatment size. The data presented (Table 3) shows that E_2_-treated tumours were more radio-resistant compared to untreated tumours. This was reflected in SGD values of 2.9±0.1 and 5.7±0.2, for E_2_-treated tumours and control, respectively.

## DISCUSSION

The physiological and clinical significance of hormonal modulation of the growth of human breast cancer cells *in vivo* are often difficult to interpret presumably as a result of the lack of host-related determinants that affect tumour behaviour. Growth of solid tumours is often regulated by interactions of endothelial cells of the tumour vasculature, tumour-infiltrating immune cells such as T-lymphocytes and macrophages, as well as the tumour cells themselves. It is known that E_2_ can increase the expression of NOS in a range of normal tissues (Weiner *et al*, 1994). In addition, some studies have shown that exposure to E_2_ may be associated with an increased incidence of breast cancer (Nenci *et al*, 1988). Our results demonstrate that long term E_2_-treatment of the putatively ER-negative human breast adenocarcinoma cell line MDA231, causes a substantial elevation in endogenous NO levels by modulating NOS expression. Significantly, E_2_-treated tumours in mice grew at a faster rate than their untreated counterparts and this is in agreement with studies carried out by others (Friedl and Jordan, 1994; Friedl *et al*, 1989). Prophylactic treatment with the NOS inhibitor, L-NAME, abolished this apparent E_2_-mediated growth stimulation. These results strongly implicate NO in mediating this increased growth response seen with the MDA 231 tumours receiving E_2_-treatment.

Confirmation of NOS expression in tumour sections was further illustrated by employing a polyclonal antibody against iNOS on tumour sections. Although positive staining for NOS occurred in all the treatment groups, it was observed that the E_2_-treatment group showed intense localization of iNOS in viable tissue, particularly in areas interfacing with necrotic tissue (i.e. regions that are hypoxic). Therefore, the actual NOS activity and consequently NO production within this tumour sub-population, could be much higher than our estimation of NOS activity using whole tumour homogenate. The observed NOS staining in tumour sections was reminiscent of that seen with *in situ* detection of iNOS mRNA expression in the human colon adenocarcinoma cell line, DLD-1 (Jenkins *et al*, 1995). These findings suggest that E_2_-stimulated increase in iNOS activity may be co-ordinately mediated by the presence of hypoxia. Hypoxia responsive elements (HREs) found within a number of genes involved in energy metabolism and angiogenesis can specifically regulate transcription response to tumour hypoxia (O'Rourke *et al*, 1997). Moreover, hypoxia has been shown to regulate endothelial mitogens such as VEGF and platelet-derived endothelial cell growth factor (PD-ECGF) in tumour cells (Minchenko *et al*, 1994). It is also known that the promoter region of the iNOS gene contains a multiplicity of consensus sequences for the binding of transcription factors, including the HRE, thus rendering the iNOS gene hypoxia- inducible (Melillo *et al*, 1995). Therefore, in tumours NOS activity can be dependent on the level of hypoxia and E_2_.

Several hypotheses have been put forward to explain the mechanistic basis of E_2_ action as a tumour growth promoter in ER-negative carcinomas. Rapid tumour growth could be accounted for as either due an increase in cell proliferation, or a decrease in cell loss (apoptosis). We propose that low and sustained NO production due to E_2_-treatment in our tumour model provides the angiogenic signal that leads to enhanced tumour cell proliferation. Human tumours in immunodeficient host animals represent a complex model; steroid hormones modulate the residual immune system, influence the endocrine milieu and alter the stroma. E_2_-treatment could enhance tumour growth by modulating the host to produce other growth factors (McGuire *et al*, 1975). Conversely, growth of cells within the tumour mass may be affected by interactions between malignant cells and the surrounding stroma. An influence of stromal fibroblasts on the growth of breast carcinoma cells has been demonstrated *in vivo* (Gleiber and Schiffman, 1984). It has also been proposed that insulin-like growth factors that act synergistically with E_2_ on breast cells, may also be produced by stromal cells (Yee *et al*, 1989; Van der Burg *et al*, 1990). Depending on its concentration, the biological redox milieu and the involvement or induction of intracellular defence mechanisms, NO can either suppress apoptosis and eventually stimulate proliferation or activate the cell death programme (Lopez-Farre *et al*, 1998). There have been studies carried out indicating that exposure to E_2_ may cause perturbation of the apoptotic pathway and may be associated with tumourigenesis (Carson and Ribeiro, 1993; Mikulski, 1994).

Another area that has received considerable attention in providing a mechanistic basis for E_2_ growth stimulation, has been the role of NK cell activity. It is widely believed that NK cells have an important role in immune surveillance against tumours (Heberman and Holden, 1979; Talmadge *et al*, 1980). A number of studies have linked suppression of NK activity with chronic administration of E_2_ (Kozlowski *et al*, 1984; Seaman *et al*, 1978; Shakhar and Ben-Eliyahu, 1998) and reduced NK activity has been associated with increased metastatic potential of different human cell lines in athymic mice (Kozlowski *et al*, 1984). The role of NK suppression is debatable and several other workers have systematically found a lack of correlation between NK cell activity and tumour growth (Fodstad *et al*, 1984, Friedl and Jordan, 1994).

Initial studies on MDA231 cells ER status led to the understanding that these cells lacked the ability to transcribe the ER gene (Weigel and deConnick, 1993), now known as ER-alpha (ER-α). More recently, another ER isoform has been identified known as ER-βε that is highly homologous to ER-α, particularly in the DNA-binding and ligand binding domains (Dechering *et al*, 2000). Variant forms of ER-β have been identified that are co-expressed with wild-type ER-β in MDA231 breast cancer cells (Fuqua *et al*, 1999; Leygue *et al*, 1999; Vladusic *et al*, 1998, 2000). In addition, available data suggests that changes in the relative expression of mRNAs for certain types of ER-α and ER-β might occur during breast tumourigenesis and tumour progression (Leygue *et al*, 1998; Pujol *et al*, 1998). It is therefore difficult to completely exclude the possibility that low and undetectable levels of ER in MDA231 tumour cells may cause growth stimulation via the classic ER-mediated pathway.

The interdependence between NO and hypoxia is believed to play an important role in controlling tumour growth and radiation response. Studies employing NO donor agents have shown that NO sensitizes hypoxic cells to ionizing radiation (Mitchell *et al*, 1993). It has been postulated that the mechanism for enhanced radiosensitivity by NO is presumably due to the rapid reaction between radiation-induced carbon centre radicals on DNA with NO thereby ‘fixing’ the damage (Howard-Flanders, 1957). It is known that the exposure of cells to low concentrations of NO can result in protection against challenges from subsequent higher concentrations (Kim *et al*, 1995). Therefore, the adaptation of hypoxic cells to spontaneous short-term NO release, such as by NO donor agents, may be different to those seen in cells that are exposed to low and sustained levels of NO generated endogenously during tumour growth. The latter situation would provide these tumour cells with a selective survival advantage over untreated cells when exposed to radiation dose. E_2_-treated tumours were significantly more radio-resistant when compared to control tumours. In conclusion, the observed alterations in the radiation response due to long term E_2_-treatment, would suggest that the level of circulating hormone should be taken into consideration in determining the timing and outcome of radiation therapy, particularly to breast cancer patients.
